# Addition of a breeding database in the Genome Database for Rosaceae

**DOI:** 10.1093/database/bat078

**Published:** 2013-11-18

**Authors:** Kate Evans, Sook Jung, Taein Lee, Lisa Brutcher, Ilhyung Cho, Cameron Peace, Dorrie Main

**Affiliations:** ^1^Washington State University Tree Fruit Research and Extension Center, 1100 N. Western Ave, Wenatchee, WA 98801; ^2^Department of Horticulture, Washington State University, Johnson Hall, Pullman WA 99164 and ^3^Department of Computer Science, Saginaw Valley State University, University Center, MI 48710, USA

## Abstract

Breeding programs produce large datasets that require efficient management systems to keep track of performance, pedigree, geographical and image-based data. With the development of DNA-based screening technologies, more breeding programs perform genotyping in addition to phenotyping for performance evaluation. The integration of breeding data with other genomic and genetic data is instrumental for the refinement of marker-assisted breeding tools, enhances genetic understanding of important crop traits and maximizes access and utility by crop breeders and allied scientists. Development of new infrastructure in the Genome Database for Rosaceae (GDR) was designed and implemented to enable secure and efficient storage, management and analysis of large datasets from the Washington State University apple breeding program and subsequently expanded to fit datasets from other Rosaceae breeders. The infrastructure was built using the software Chado and Drupal, making use of the Natural Diversity module to accommodate large-scale phenotypic and genotypic data. Breeders can search accessions within the GDR to identify individuals with specific trait combinations. Results from *Search by Parentage* lists individuals with parents in common and results from *Individual Variety* pages link to all data available on each chosen individual including pedigree, phenotypic and genotypic information. Genotypic data are searchable by markers and alleles; results are linked to other pages in the GDR to enable the user to access tools such as GBrowse and CMap. This breeding database provides users with the opportunity to search datasets in a fully targeted manner and retrieve and compare performance data from multiple selections, years and sites, and to output the data needed for variety release publications and patent applications. The breeding database facilitates efficient program management. Storing publicly available breeding data in a database together with genomic and genetic data will further accelerate the cross-utilization of diverse data types by researchers from various disciplines.

Database URL: http://www.rosaceae.org/breeders_toolbox

## Background

Breeding programs generate large volumes of data and, with at least 18 years from seed to release being typical for a tree fruit crop like apple (*Malus* × *domestica* Borkh.), an efficient management system for keeping track of the data collected each year is essential for a program to be successful. Current systems range from use of a field book, through commonly available software such as Microsoft Excel and Access, to dedicated commercial software. Although commercial database packages are available for plant breeders, for example, Agrobase® (Agronomix, Inc., Winnipeg, Canada), these are predominantly focused on annual agricultural crops rather than perennial and clonally propagated crops such as apple. In addition, breeders are now using DNA information, which makes their data management needs more complex.

The Washington State University apple breeding program (WABP) is an example of a modern breeding program requiring efficient management of a large data volume from diverse sources. This program produces, on average, 20 000 genetically different seedlings a year (1). These seedlings go through several rounds of selection until ∼5000 are finally planted in the orchard for fruit evaluation. Once the trees are established and productive, fruit is sampled over several weeks to ensure the stage of optimum maturity is covered. Fruit quality descriptions of parents and selections form the majority of data collected. Samples from the same selection are collected weekly for up to 4 weeks depending on the amount of fruit available and are tested at harvest, and after 2 and 4 months of regular atmosphere cold storage. Sample labels allow the breeder to distinguish and compare fruit data from the different harvest dates and storage periods. Some key performance traits can be measured instrumentally, for example, fruit texture using a Mohr® DigiTest (Mohr and Associates, Richland, Washington, USA), titratable acidity and soluble solids; however, other traits are scored on a sensory scale. Seedlings judged by the breeder to be superior are vegetatively propagated and planted at several different sites to allow investigation of environmental effects with different growing conditions. Fruit and tree growth are evaluated over several years before the decision is made to release or reject a variety. Although much of this pre-release breeding data must remain confidential, the program also produces data that can be made publicly available. With the increased application in the WABP of DNA-based tools and information for more efficient planning of crosses and for screening of seedlings, the challenge of data management has become more difficult. With this volume of data from many individual trees over multiple years and sites, it is imperative that the breeder not only has confidence in the data management system but also has the ability to interrogate the system to readily access required data and extract summary statistics for timely action.

The Genome Database for Rosaceae (GDR) (2) collaborated with the WABP to build an infrastructure to integrate breeding data with the existing genomic and genetic data of the Rosaceae crop family. This infrastructure has now also been adopted for the public datasets of the USDA-NIFA SCRI-funded RosBREED project (3). The GDR is the central repository for genomic and genetic data of Rosaceae crops. Available resources in the GDR include the annotated whole genome sequences of apple (4), peach (5) and woodland strawberry (6), annotated EST sequences, genetic maps, genetically mapped traits and genotypic diversity data. The genome browser, GBrowse (7, 8) is available for genome sequence and annotation data visualization, and CMap (9), an integrated and comparative map viewer, can be used to query and view the genetic map data. Predicted genes, ESTs, BACs, markers, traits and polymorphism data can be queried through simple and advanced search interfaces. From each type of data page, users can access other GDR data pages and graphical interfaces for exploration of integrated data, facilitating the use of data across scientific disciplines.

This article reports a new breeding database in the GDR that provides a secure and private database management system for breeders that is fully integrated with GDR data types and with integrated analytical capabilities, to enable use of public marker, trait and genomics data, as well as to provide the same analytical capabilities to public users accessing publicly available breeding data.

## Construction and content

### Data

The breeding data that are stored in the GDR include integrated phenotypic data, genotypic data, germplasm and pedigree data from the WABP. In breeding programs, parental information forms the core dataset, as planning optimal crosses requires detailed data perusal and intensive value-added analyses. Field location is included, as it is essential that all germplasm can be easily tracked physically. Each new clone of vegetatively propagated selections is distinguished within the database with its own unique identity and performance data. The database provides the opportunity to compare performance of selections between years and across sites. With a crop like apple in which appearance is a critical selection criterion, it is also vital to have the capacity to upload images and add text-based comments. For the WABP, fruit images are also stored in the database. Genotypic data for microsatellite markers are also stored. Large-scale SNP genotypic data (10) will be included in the near future. The phenotypic and genotypic data have been integrated with data in the GDR that include details of the genetic markers that have been used in genotyping, such as primers, associated sequences, polymerase chain reaction conditions, genetic locations, literature reference and expert contact information. The GDR contains secured private breeding data as well as public data. The current public apple dataset includes 125 504 phenotypic measurements divided between 95 trait descriptors of 91 varieties plus 552 genotypic data points. The same descriptors are used for the private set; 572 varieties and selections are described by 284 552 phenotypic measurements and 472 genotypic data points. In addition, breeding data from the RosBREED project (3) have been added recently, including both public and private data for apple (170 trait descriptors of 557 and 664 individuals, respectively), peach (43 trait descriptors of 576 and 453 individuals, respectively), strawberry (76 trait descriptors of 695 and 188 individuals, respectively), sweet cherry (39 trait descriptors of 199 and 237 individuals, respectively) and tart cherry (58 trait descriptors of 335 and 72 individuals, respectively). The total numbers of RosBREED phenotypic measurements and genotypic data points are 606 411 and 7498, respectively.

### Database schema

To store the breeding data, we have used the newly developed Natural Diversity (ND) module (11) along with other related modules, such as Stock, Phenotype and Genetic modules of Chado (12), the generic schema for biological databases. The varieties, and their clones and samples, are stored in the *stock* table of the Stock module. Their pedigrees are stored in the *stock_relationship* table of the Stock module. The core of the ND module is the *nd_experiment* table where any type of an event that involves an entry in the *stock* table, such as addition of phenotypic or genotypic data, inclusion of descendants from crossing or creation of a new clone from vegetative propagation, can be stored. Each row of *nd_experiment* has a link to site information associated with the data point according to the geographical location in which it was conducted, stored in the *nd_geolocation* table. Related rows of the *nd_experiment* table can be combined into a project, using the project table in Chado, so that breeders can store hierarchical datasets. The phenotypic and genotypic data are stored in the Phenotype and Genetic modules, which are connected to the Stock module via the ND module. Following the characteristics of the Chado design, we use controlled vocabulary, stored in the controlled vocabulary module, to describe the types of experiments, phenotypic descriptors, stock relationships and other metadata. DNA marker information is stored in the Sequence module, which is linked to the Genetic module. The schema of the ND module is available in (11), and descriptions of tables of further modules in Chado are available at http://gmod.org/wiki/Chado.

### Data uploading

Data templates in a Microsoft Excel file have been created for breeders to record their data. The template is composed of several sheets to record the information on variety, cross, phenotypic descriptors and phenotyping and genotyping efforts. The template is flexible so that breeders can directly use the output files from data-generating equipment such as fruit analysis equipment and field dataloggers. This connectivity allows minimal data handling and thus reduces the opportunity for errors. A data-uploading script is written in Perl for uploading to the Chado database. All the data submitted to the database by the breeder are checked by curators and uploaded using the in-house Perl script. If the template is filled in following the instructions, little further curational editing is needed.

### Web interface construction

As with the other interfaces in the GDR, we have used Drupal, the popular Content Management System (CMS), to construct web pages for the breeding database. CMSs help simplify installation and management of Web sites, development of web pages and web content changes. Drupal is one of the most popular CMSs because it is open source and maintains a repository of extensive user-contributed modules on the Drupal development Web site. These modules are freely available and can be used to easily extend and customize Web site functionality. Categories of modules include commerce/advertising, community, content access control, file management and user management, to name a few. We have used these modules extensively to build the breeding database efficiently. One of the modules called *Organic Group*, allows users to create and manage their own *groups.* A group is a collection of users, and each group can be managed by a non-technical manager, and have subscribers and a homepage where subscribers can communicate among themselves. Use of the *Organic Group* module allows each breeder to have their own homepage with their own subscribers. This feature is also used for data access control. Members of a group are given permission to access their private data. All the breeding data are stored in Chado, and the user information is stored in Drupal tables. In Chado, a materialized view was built for private data from each Organic group. A Drupal function returns all the Organic groups available to the logged in user and is used in the breeding database to restrict access of materialized views. Public users who do not have a Drupal account (anonymous users) can access the public portion of breeding data in the breeding database. Currently, all the data of public varieties are available to public users. Private varieties or breeding selections are tagged in the database and all of their data can be accessed only by those users who belong to the appropriate Organic Group. The schema also allows tagging of individual measurements and storage of some of the measurement data in the protected materialized views even if the variety information and some of the measurement data are publicly available should that data require protection in the future. Various web pages where users can browse, query, view and/or download the queried results are developed using PHP: Hypertext Preprocessor.

## Utility

The breeding database in the GDR provides a breeder with an easy-to-use customizable resource that allows interrogation and retrieval of breeding data using various search options, as well as controlled access to private data depending on the user’s privileges. In addition, it allows the breeders to create individual homepages easily for which they can add their own web pages and upload documents to facilitate communication and document-sharing among members of the same breeding team. The homepage for the breeding database displays hyperlinks for all the interfaces. The interface is intuitive and comes with an extensive tutorial including screenshots to help users explore the data and database functionalities. The major web interfaces and tools of the breeding database in the GDR are described later.

### Browsing and searching functionality

**Figure 1.**
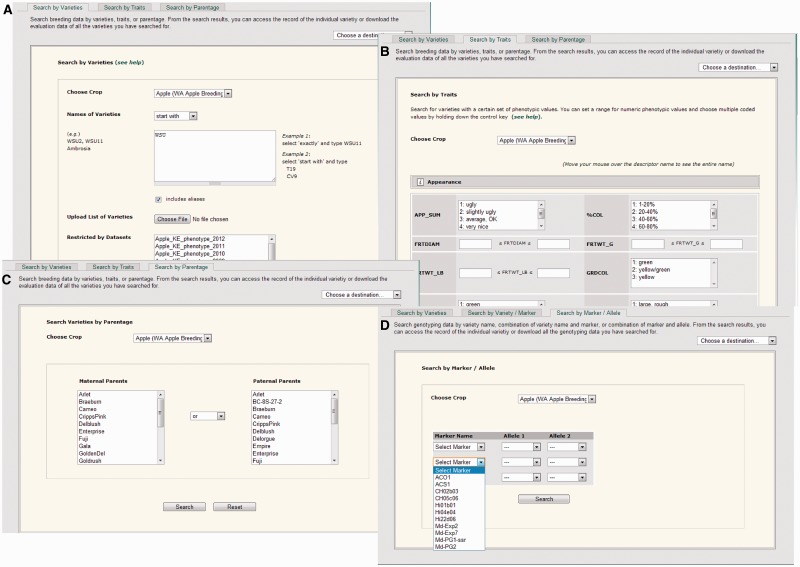
Search pages for phenotypic and genotypic data. Users can search varieties with phenotypic data using variety names (**A**), trait values (**B**) or parentage (**C**). Varieties with genotypic data can be searched by variety name, the combination of variety name and marker or by alleles (**D**).

Breeders can browse datasets in their database to view varieties across all or within particular datasets and download all or specific breeding data associated with those varieties. Breeders can group breeding data using categories such as year, site or data type in the data template, and the dataset page displays specified datasets. The breeding database includes comprehensive search pages for phenotypic and genotypic data. Users can search by variety names and traits or by parentage (Figure 1). When searching by variety names, users can enter a specific variety name(s) or upload a list. In the ‘Search by Trait page’, users can choose individual traits or groups of traits within specified trait thresholds. Trait descriptors in the page are organized into groups, such as ‘Appearance’ and ‘Production’ that have been previously specified by the data provider (breeder), so that breeders can easily navigate among traits. Inclusion of pedigree data allows breeders to search for any families or individuals with a particular parent or combination of parents in the ‘Search by Parentage’ page. Varieties with genotypic data can be searched by variety, by variety and marker or by marker and allele, providing the breeder with considerable flexibility in approach (Figure 1). Further information about each marker can be accessed via hyperlinks to the genomic and genetic data section of the GDR, enabling ready access to protocols, allelic variation, sequence data, physical map position and genetic map position. For example, from the *Marker* page users can go to CMap, the comparative map viewer, to view other closely linked markers in various genetic maps. If an apple genetic marker is anchored to the apple whole genome sequence, the *Marker* page provides a link to GBrowse, where users can browse other features at the locus such as genes, ESTs, other markers, primers, trait loci and homologs in related species.

### Search results for accessing individual records or downloading data

**Figure 2.**
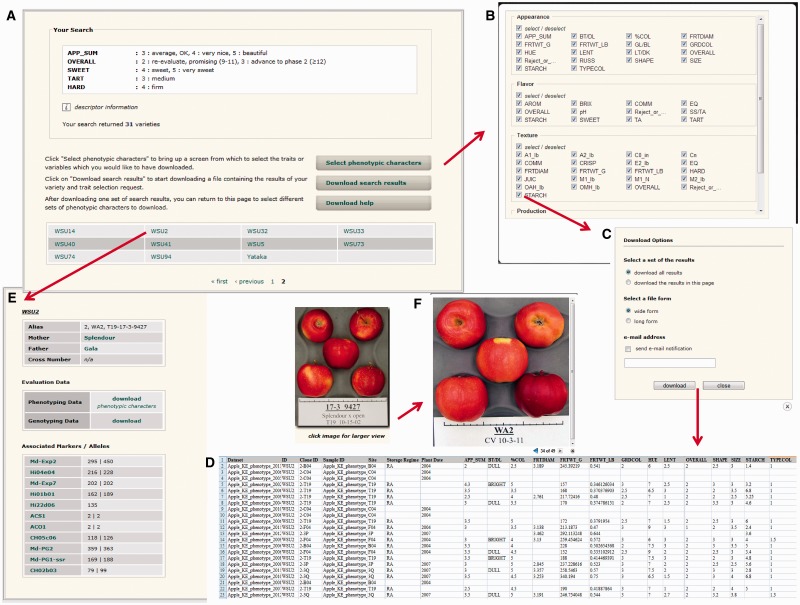
Interfaces to view and download data resulting from a search in the breeding database. (**A**) *Search Result* page that shows the search categories, a list of varieties meeting specified criteria and hyperlinks to download the results. (**B**) Pop-up window where users can choose traits to include in the downloaded file. (**C**) Pop-up window where users can choose download options such as format of the downloaded file, data (in the current page or all pages) and download method (via email or online). (**D**) An example of the data downloaded into Excel format. (**E**) *Individual Variety* page that can be accessed from the *Search Result* page. The page shows the pedigree information, photos if available (for example, F), associated alleles and links to download phenotypic and genotypic data.

Once the list of varieties is generated through the browsing and searching pages, users can download data for multiple varieties or go to the ‘Variety’ page to access individual records (Figure 2). When downloading phenotypic data from the ‘Search Result’ page or the ‘Variety’ page, users can choose the traits they want to include in the downloaded dataset (Figure 2B). Users can also choose the format of the downloaded file, long or wide form, to suit their subsequent needs (Figure 2C). In wide form, all the trait values of one sample with each trait descriptor as a column heading are shown in the same row. In long form, each row shows one trait value of one sample. Users can choose to download online or via email (Figure 2C). When the option for downloading online is chosen for a large dataset, a pop-up window appears to suggest downloading via email. Users with a GDR account can access the *‘*User Account’ page to view the progress of the download. The ‘User Account’ page also lists recently downloaded tasks.

The ‘Variety’ page shows any synonyms and pedigree information such as the mother, father and cross number, if applicable (Figure 2E). The ‘Variety’ page also shows photos if available. Users can click the image to zoom in and view any other images for the variety (Figure 2F). Also displayed is the list of all markers for which genotypic data are available for the variety and the alleles of those markers recorded for that variety. All phenotypic and genotypic data of a variety can also be downloaded from the *Variety* page.

### Use cases

An example of a search for breeding data would be to identify individual seedlings with specific levels of several traits. Using the ‘Search by Traits’ page, the user can set trait thresholds using a combination of appearance, flavor, texture and production traits. A search in the public WABP database might be for ‘Appearance’ = 3–5 (average to beautiful), ‘Overall score’ = 2–3 (promising and advance to phase 2), ‘Sweetness’ = 4–5 (sweet and very sweet), ‘Tartness’ = 3 (medium acid) and ‘Hardness’ = 4 (firm). Such a search currently results in a list of 31 individuals (Figure 2A), including both seedling and reference varieties. Each individual will connect to its own ‘Variety Detail’ page (Figure 2E).

**Figure 3.**
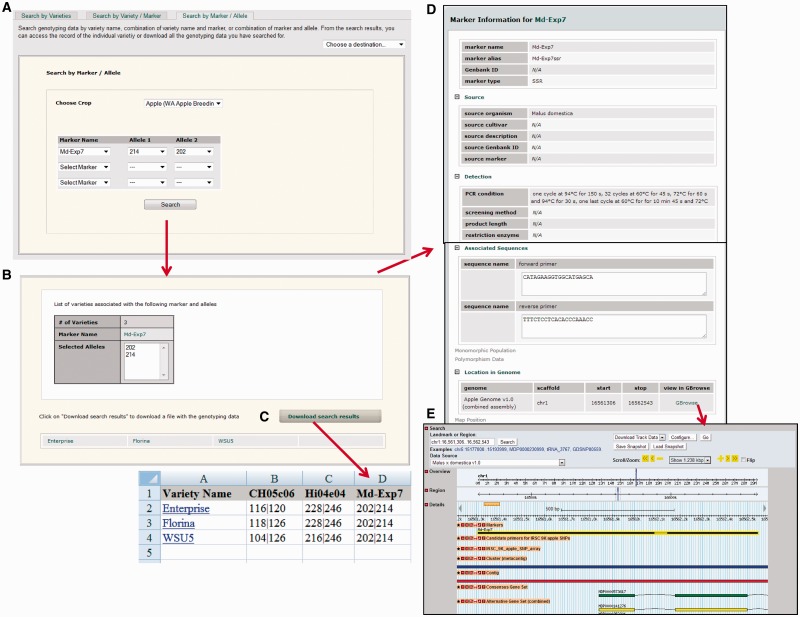
Interfaces to view and download genotypic data resulting from a search in the breeding database. (**A**) *Search by Marker/Allele* page that shows the crop and marker allele(s) chosen. (**B**) List of individuals associated with the marker allele(s) chosen. (**C**) An example of the data downloaded into Excel format. (**D**) An example of the marker information available by clicking on the marker name. (**E**) GBrowse view of the example marker showing its position in the genome and other associated information.

Advances in integrating genotypic and phenotypic data are leading rapidly to the identification of favorable and unfavorable alleles for particular markers (13,14). ‘Search by Marker/Allele’ allows the breeder to list the germplasm individuals within the database with a specific allele, thus enabling the breeder to identify those individuals as possible parents or to avoid them for future crossing. The *Md-Exp7* simple sequence repeat (SSR) marker is linked to resistance to apple scab (*Venturia inaequalis*) and is putatively associated with flesh firmness differences (13). Apple varieties or selections with the *Md-Exp7* allele 214 are likely to be resistant to scab. By using the *Search by Marker/Allele* page (Figure 3A), choosing *Md-Exp7* and allele 214 (together with allele 202 in this example) in public WABP data, three individuals are listed with this desirable allele (Figure 3B). Users can go to the hyperlinked marker page (Figure 3D) to view details such as primers, genetic map position, anchored genome position and publication and follow the link to view the marker in the genome browser, GBrowse (Figure 3E) to explore genomic features near the marker. Connecting to the appropriate *Variety Detail* page provides further information including the other allele of *Md-Exp7* for a particular variety or seedling as well as genotypic data for other SSRs.

## Discussion

The new breeding database in the GDR is far more than just a storage database for breeding data. This database provides users with the opportunity to search integrated breeding-genetic-genomic datasets in a fully targeted manner and retrieve and compare performance data from multiple varieties and seedlings, years and sites, streamlining selection decisions and outputting the type of data needed for variety release publications and patent applications. The database enables more efficient program management. The integration of breeding data in the GDR can be useful to other biologists such as quantitative geneticists, molecular geneticists and molecular biologists because it allows users to interrogate relationships among varieties, alleles and traits. The fact that the breeding data are stored in the database together with genomic and genetic data is expected to further accelerate data use by researchers from various disciplines. The breeding database will allow researchers’ comparisons of allelic variation with trait variation, to choose appropriate sets of varieties for genetic dissection of traits and for further examination of allelic variation, to study environmental effects on performance and to evaluate the efficacy of genetic tests. Developing the breeding database within the GDR provides an excellent opportunity for easy access to publicly available data that could be used to enhance breeders’ selection ability with newly developed genetic tests or expand breeders’ private analyzable datasets with public breeding data from projects such as RosBREED (3).

The breeding database is built using open source software, Chado and Drupal. Using open source software provides many benefits, including cost savings and flexibility (15). Being open source enabled collaboration among Chado database developers from several biological systems to add the new ND module to accommodate large-scale phenotypic and genotypic data (11). Chado’s highly flexible ontology-driven modularity enables the same schema to be used in projects with widely different metadata. Metadata can be modified or added as new data types become available. Using Drupal streamlined building of the breeding database content and the implementation of data access control and functionality for creating individual group home pages. As more functionality is developed in Chado and Drupal, we will be able to improve the interface, developing more functionality to meet breeding program needs.

Several new developments are planned to improve the functionality for the breeder. Currently, data are supplied from the breeder in an Excel template and are uploaded by the database team; any subsequent edits have to be conducted by the database team rather than by the breeder. New web interfaces will provide data uploading and editing capability to the breeder. Further web interfaces to return search results directly to the computer screen, in addition to the downloadable Excel file, and to allow searches to be saved, retrieved, edited and compared are also planned. More publicly available trait and marker data will be added to enhance the datasets and likely suggest further useful functionalities. As a part of RosBREED project, software modules to aid development of DNA tests from reported QTLs through to their use in breeding decisions are also under development.

## Conclusions

We have built a breeding database using open source software that enables users to access publicly available Rosaceae breeding data through the GDR. In addition, the database provides a secure area for storage and management of private breeding data. Sufficient functionality has been included to enable the user to search data by variety, trait, parentage or genotype. Additional functionality has been achieved by connecting the breeding database through the GDR to other software such as GBrowse and CMap.

## Availability

Public data from the WABP and RosBREED project are currently available from the GDR (www.rosaceae.org/breeders_toolbox).
